# Nonapnea Sleep Disorders in Patients Younger than 65 Years Are Significantly Associated with CKD: A Nationwide Population-Based Study

**DOI:** 10.1371/journal.pone.0140401

**Published:** 2015-10-14

**Authors:** Hugo You-Hsien Lin, Chi-Chih Hung, Yu-Han Chang, Ming-Yen Lin, Ming-Yu Yang, Shih-Shin Liang, Wangta Liu, Hung-Chun Chen, Shang-Jyh Hwang

**Affiliations:** 1 Division of Nephrology, Department of Internal Medicine, Kaohsiung Medical University Hospital, Kaohsiung Medical University, Kaohsiung, Taiwan; 2 Department of Internal Medicine, Kaohsiung Municipal Ta-Tung Hospital, Kaohsiung Medical University, Kaohsiung, Taiwan; 3 Graduate Institute of Medicine, College of Medicine, Kaohsiung Medical University, Kaohsiung, Taiwan; 4 Graduate Institute of Clinical Medical Sciences, College of Medicine, Chang Gung University, Tao-Yuan, Taiwan; 5 Department of Biotechnology, College of Life Science, Kaohsiung Medical University, Kaohsiung, Taiwan; 6 Faculty of Renal Care, College of Medicine, Kaohsiung Medical University, Kaohsiung, Taiwan; 7 Institute of Population Sciences, National Health Research Institutes, Miaoli, Taiwan; National Health Research Institutes, TAIWAN

## Abstract

**Background:**

Nonapnea sleep disorders (NASD) and sleep-related problems are associated with poor health outcomes. However, the association between NASD and the development and prognosis of chronic kidney disease (CKD) has not been investigated thoroughly. We explored the association between CKD and NASD in Taiwan.

**Methods:**

We conducted a population-based study using the Taiwan National Health Insurance database with1,000,000 representative data for the period from January 1, 2000 to December 31, 2009. We investigated the incidence and risk of CKD in 7,006 newly diagnosed NASD cases compared with 21,018 people without NASD matched according to age, sex, index year, urbanization, region, and monthly income at a 1:3 ratio.

**Results:**

The subsequent risk of CKD was 1.48-foldhigher in the NASD cohort than in the control cohort (95% confidence interval [CI] = 1.26–1.73, p< 0.001). Men, older age, type 2 diabetes mellitus, and gout were significant factors associated with the increased risk of CKD (p< 0.001). Among different types of NASDs, patients with insomnia had a 52% increased risk of developing CKD (95%CI = 1.23–1.84; P<0.01), whereas patients with sleep disturbance had a 49%increased risk of subsequent CKD (95% CI = 1.19–1.87; P<0.001). Younger women (aged < 65 years) were at a high risk of CKD with NASD (adjusted hazard ratio, [HR] = 1.81; 95% CI = 1.35–2.40, p< 0.001).

**Conclusions:**

In this nationwide population-based cohort study, patients with NASD, particularly men of all ages and women aged younger than 65 years, were at high risk of CKD.

## Introduction

The prevalence and incidence of chronic kidney disease (CKD) have increased over the past decade and this trend seems likely to continue. Thus, CKD is a growing public health problem worldwide [1,2]. Evaluating the risk factors for CKD is crucial because of the high all-cause mortality and cardiovascular disease associated with the disease [3]. Although CKD is mainly caused by hypertension and type 2 diabetes mellitus (DM)[4], the factors responsible for its development or progression have yet to be investigated. Hyperuricemia, dyslipidemia, obesity, and inflammation are other risk factors for CKD but are only partially responsible for the individual differences [5–8]. Sleep disorders are common conditions characterized by difficulty in initiating or maintaining sleep, accompanied by symptoms such as irritability or fatigue during wakefulness. The prevalence of nonapnea sleep disorders (NASD) in the general population is approximately 20%, of which approximately 50% are chronic cases that mostly remain undiagnosed [9]. The prevalence of sleep disorders and sleep-related problems (leg symptoms and nocturia) are higher in patients with CKD compared with patients without CKD [10]. Among sleep disorders, such as obstructive sleep apnea (OSA),are risk factors for cardiovascular disease (CVD), hypertension, stroke, CKD, and mortality[11,12]. The underlying pathogenesis of OSA comprises intermittent hypoxia, activated sympathetic nerve activity, systemic inflammation, and oxidative stress [13–15]. We evaluated the association of NASD with the risk of CKD in Taiwan, and the study data were obtained from Taiwan’s National Health Insurance Research Database (NHIRD).

## Methods

### Study Population

The National Health Insurance (NHI) program in Taiwan is a nationwide, compulsory, and comprehensive insurance system initiated in 1995 and established by the Bureau of National Health Insurance of the Department of Health. The NHI provides health care to 99% of the 23.74 million residents of Taiwan and is contracted with 97% of Taiwanese hospitals and clinics[16]. The NHIRD, one of the largest databases worldwide, is released for research and contains the claims data on one million people systematically selected from all the insurants. The NHIRD includes encrypted patient identification numbers, medical facility registries, details of ambulatory care, inpatient orders, dental services, prescribed drugs, and physicians providing services. The disorders diagnosed are coded according to the International Classification of Diseases, Ninth Revision, Clinical Modification (ICD-9-CM). This cohort study was approved by the Institutional Review Board of Kaohsiung Medical University (KMUH-IRB-EXEMPT-20140059).

#### Study Participants

**Fig 1:**
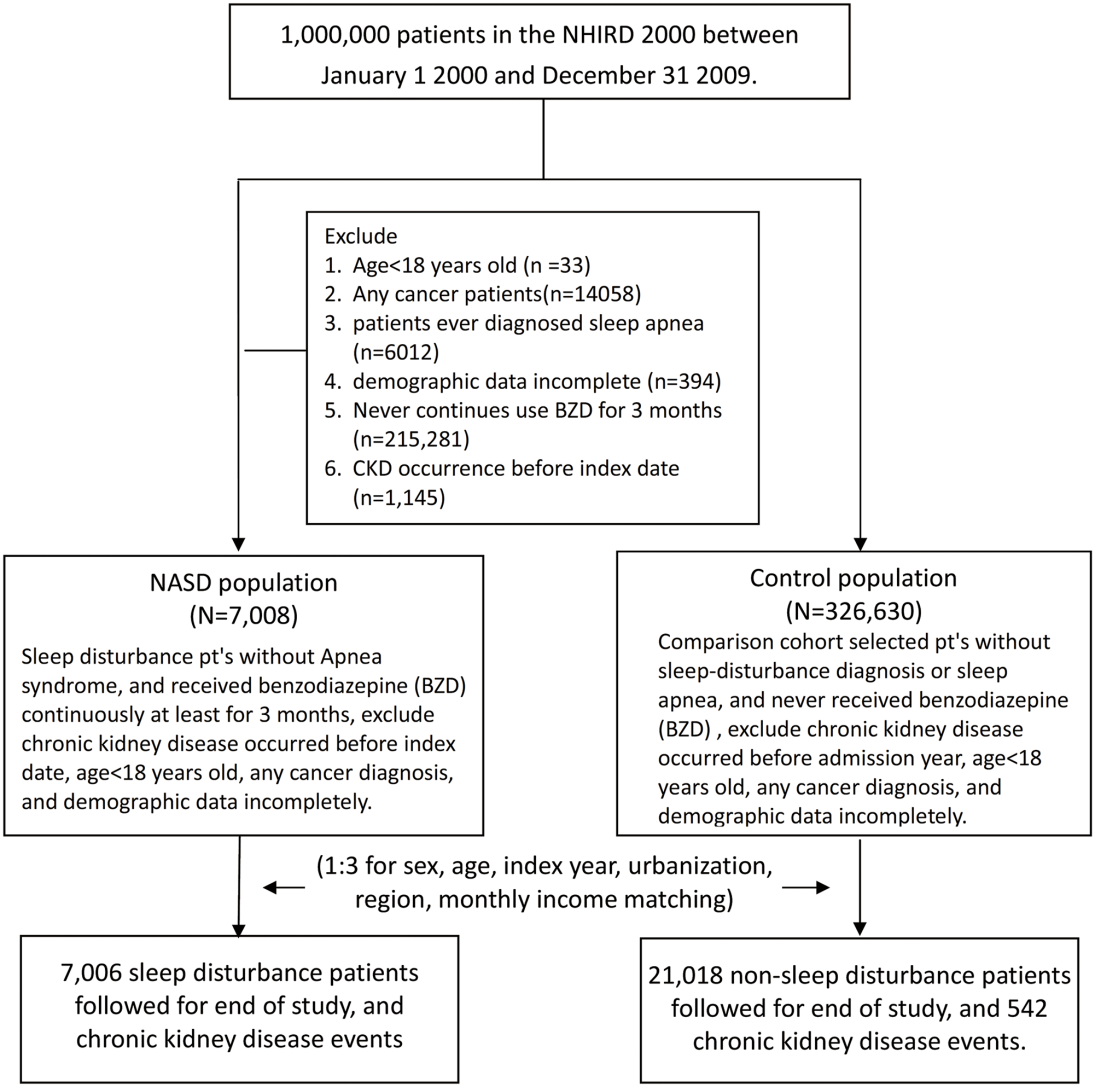
Flow diagram of the study population.

We conducted a retrospective cohort study. Patients with sleep disorders, including nonorganic sleep disorders and sleep disturbances (ICD-9-CM codes 307.4x, 780.5x, 333.94, 347, 327.3x)newly diagnosed by physicians and who used benzodiazepine (BZD) before bedtime for at least 3 months between January 1,2000 and December 31, 2010 according to the NHIRD records comprised the NASD cohort. The date of first NASD diagnosis was considered the index date. We excluded patients aged younger than 18 years, having sleep apnea syndrome (ICD-9-CMcodes 780.51, 780.53, 780.57 and 327.23), and history of CKD (ICD-9-CM code 585) before the index date. NASD were classified into insomnia (ICD-9-CM code 780.52), sleep disturbance(ICD-9-CM code 780.5), unspecified sleep disturbance (ICD-9-CM code 780.50), unspecified hypersomnia (ICD-9-CM code780.54), unspecified disruptions of 24-h sleep–wake cycle (ICD-9-CM code 780.55), dysfunctions associated with sleep stages or arousal from sleep (ICD-9-CM code 780.56), unspecified sleep-related movement disorder (ICD-9-CM code 780.58), other sleep disturbance (ICD-9-CM code 780.59), specific sleep disorders of nonorganic origin (ICD-9-CM code 307.4),restless legs syndrome (ICD-9-CM code 333.94), cataplexy and narcolepsy (ICD-9-CM code 347), and circadian rhythm sleep disorder (ICD-9-CM code 327.3x)[17]. The control cohort comprised randomly selected patients without a history of sleep disorders, and CKD, who were frequency matched according to sex, age, index year, urbanization, region, and monthly income. A matching procedure was applied to enhance the comparison between the NASD and control cohorts. The index year was defined as the year of NASD diagnosis for the NASD cohort, whereas it was the year of an outpatient visit for the control cohort. Age was calculated from the date of birth to the date of NASD diagnosis for the NASD cohort and from the date of birth to the date of the outpatient visit for the control cohort. The participants in the control cohort were matched with the NASD cohort at a 3:1 ratio. The follow-up period started from the date of entering the study cohort to the date of CKD event, censoring, or December 31, 2010 (Fig 1).

#### Outcome Measures

In Taiwan, patients with end-stage renal disease (ESRD) requiring dialysis can apply for a catastrophic illness card. Cardholders are exempt from the cost sharing required by the NHI program. Patients with ESRD were defined as patients who had received a catastrophic illness card for dialysis and claimed for hemodialysis or peritoneal dialysis for at least 3 months (ICD-9-CM code 585). Patients with CKD were defined as patients without ESRD who were hospitalized at least once or had 3 or more outpatient visits, in which one or more of the following ICD-9-CM diagnostic codes were used: 585–589, 250.4, 274.1, 283.11, 403.x1, 404.x2, 404.x3, 440.1, 442.1, 447.3, 572.4, 642.1x, 646.2x, and 794.4. The person-years of follow-up of the patients were estimated from the index date to the CKD diagnosis date, censoring caused by death during hospitalization, loss to follow-up, withdrawal from the insurance system, or the end of December 31, 2010. The follow-up period of CKD prognosis started from the CKD date to the end point of the study. The comorbidities included in our study were hypertension(ICD-9-CM codes 401–405), DM (ICD-9-CM code250), hyperlipidemia (ICD-9-CM code 272), CVD (ICD-9-CM codes 410, 412, 428), cerebrovascular diseases (ICD-9-CM codes 430–438), liver disease (ICD-9-CM codes 571–572, 456.0–456.2), gout (ICD-9-CM code 274.x), obesity (ICD-9-CM code 278.x), and depression (ICD-9-CM codes 296.2, 296.3).

### Validation

We validated the ICD-9-CM codes for the identification of NASD and CKD by analyzing the medical records (charts) of 200 patients, who had NASD ICD-9-CM code780.52, 780.5, 780.50, 780.54, 780.55, 780.56, 780.58 and 780.59; CKD ICD-9-CM code 585 from the inpatient and outpatient claims database between January 2008 and December2010 in Kaohsiung Municipal Ta-Tung Hospital, which is a regional teaching hospital in Taiwan. The contents of this database were similar to those of the NHIRD. The clinical diagnosis of NASD was ascertained by psychiatrists and neurologists. Clinical diagnosis of CKD was determined according to the estimated glomerular filtration rate < 60 mL/min/1.73 m2 for more than 3 months. Positive predictive values of both diseases were estimated. There are 184 cases confirmed the diagnosis with NASD and 196 cases confirmed the diagnosis with CKD. The positive predictive value (PPV) of NASD and CKD are 0.92 and 0.98, separately.

### Statistical Analysis

An independent *t* test, chi-square test, or Fisher’s exact test was employed for comparing the distribution of risk factors between the NASD and control cohorts. Cox proportional hazard regression analyses were conducted to calculate the crude and adjusted hazard ratios (HRs) for the risk of CKD. Multiple Cox proportional hazard regression analyses were performed after adjustment for sex, age, and any history of hypertension, DM, hyperlipidemia, cerebrovascular disease, CVD, liver disease, gout, obesity, and depression. Kaplan–Meier curves were applied to estimate the probability of CKD onset, and the log-rank or Gehan–Breslow–Wilcoxon test was used to examine the differences among groups. Statistical analyses were performed using SAS 9.3 software (SAS Institute, Inc., Cary, NC, USA). Statistical significance was set at p< 0.05.

## Results

### Baseline characteristics of the study cohorts

For the period 2000–2010, we identified and selected7,006 and 21,018 patients as the NASD and the control cohorts, respectively. The mean age of the patients was 53.91 ± 16.45 years, and 56.2% of patients in the NASD cohort were women (Table 1). The distributions of age, sex, index year, urbanization, resident region, and monthly income were similar between the 2 cohorts. Compared with the control cohort, patients in the NASD cohort had more medical visits in 1 year before the index date (p<0.001) than did those in the control cohort. Furthermore, the patients in the NASD cohort were more likely to have comorbidities such as hypertension, DM, hyperlipidemia, CVD, cerebrovascular disease, liver disease, gout, obesity, and depression before the index date compared with those in the control cohort (p< 0.005).

**Table 1:** Demographic characteristics between the NASD cohort and control cohort (N = 28,024).

	NASD cohort (n = 7,006)		Control cohort (n = 21,018)		p value
Age(Mean±SD)	53.91	(±16.45)	53.62	(±16.81)	0.208
≦40	1688	(24.1)	5069	(24.1)	
41–65	3466	(49.5)	10457	(49.8)	
>65	1852	(26.4)	5492	(26.1)	
Sex(%)					
Female	3936	(56.2)	11808	(56.2)	1.000
Male	3070	(43.8)	9210	(43.8)	
Index Year					
2000–2003	3058	(43.7)	9171	(43.6)	0.999
2004–2006	2280	(32.5)	6841	(32.6)	
2007–2009	1668	(23.8)	5006	(23.8)	
Urbanization					
Urban	2521	(36.0)	7563	(36.0)	1.000
Suburban	2917	(41.6)	8750	(41.6)	
Rural	1568	(22.4)	4705	(22.4)	
Region					
Northern	3794	(54.1)	11382	(54.1)	1.000
Central	1680	(24.0)	5036	(24.0)	
Southern	1286	(18.4)	3859	(18.4)	
Eastern	246	(3.5)	741	(3.5)	
Monthly Income					
<15,000	2564	(36.6)	7697	(36.6)	0.999
15,000–29,999	3260	(46.5)	9780	(46.5)	
≧30,000	1182	(16.9)	3541	(16.9)	
Visit Ambulatory average frequency					
Mean±SD	0.23	(±0.85)	0.11	(±0.44)	<0.001
Comorbidities(%)					
Hypertension	1411	(20.1)	2191	(10.4)	<0.001
DM	570	(8.1)	1004	(4.8)	<0.001
Hyperlipidemia	785	(11.2)	1242	(5.9)	<0.001
Cerebral vascular disease	596	(8.5)	857	(4.1)	<0.001
CVD	277	(4.0)	506	(2.4)	<0.001
Liver disease	1080	(15.4)	2124	(10.1)	<0.001
Gout	387	(5.5)	734	(3.5)	<0.001
Obesity	30	(0.4)	42	(0.2)	<0.001
Depression	909	(13.0)	467	(2.2)	<0.001

Abbreviation: NASD: nonapnea sleep disorders; SD: standard deviation; DM: type 2 Diabetes Mellitus; CVD: cardiovascular disease; COPD: chronic pulmonary disease.

### Association of CKD with NASD according to age, sex, and comorbidities

During the follow-up period, the overall incidence of CKD was higher in the NASD cohort than in the control cohort (6.42 vs. 3.95 per 10,000 person-years, Table 2). After adjustment for the covariates of baseline characteristics and comorbidities, the risk of CKD was significant for patients with NASD(HR = 1.48; 95% CI = 1.26–1.73, p< 0.001). Men had a higher incidence of CKD in both cohorts and a 41%increase in the risk of CKD(p<0.001). The incidence of CKD increased with age(p< 0.001). The age-specific relative risks of CKD were higher in middle- and old-aged adults than in young adults(aged 41–65 years, HR = 2.30, 95% CI = 1.76–3.01,p<0.001; aged older than 65years, HR = 4.08, 95% CI = 3.12–5.35, p<0.001). The incidence of CKD increased for patients with preexisting comorbidities before the index date. The comorbidities included DM (HR = 1.70, 95% CI = 1.36–2.12,p< 0.001) and gout (HR = 1.84, 95% CI = 1.44–2.36, p< 0.001).

**Table 2:** Cox proportional hazards regression model for risk of CKD between the NSAD cohort and control cohort (N = 28,024).

	Crude			Adjusted		
	HR	(95% CI)	p value	HR	(95% CI)	p value
NASD						
No	Ref.			Ref.		
Yes	1.62	(1.39–1.88)	<0.001	1.48	(1.26–1.73)	<0.001
Gender						
Female	Ref.			Ref.		
Male	1.45	(1.26–1.67)	<0.001	1.41	(1.22–1.64)	<0.001
Age						
≦40	Ref.			Ref.		
41–65	2.46	(1.90–3.19)	<0.001	2.30	(1.76–3.01)	<0.001
>65	4.86	(3.75–6.31)	<0.001	4.08	(3.12–5.35)	<0.001
Urbanization						
Rural	Ref.			Ref.		
Suburban	1.10	(0.90–1.33)	0.361	1.16	(0.92–1.47)	0.211
Urban	1.12	(0.92–1.35)	0.264	1.21	(0.99–1.49)	0.059
Region						
Northern	Ref.			Ref.		
Central	0.93	(0.78–1.11)	0.446	0.95	(0.79–1.16)	0.632
Southern and eastern	0.93	(0.77–1.12)	0.424	0.94	(0.76–1.17)	0.577
Monthly Income						
<15,000	Ref.			Ref.		
15,000–29,999	0.88	(0.75–1.03)	0.102	0.96	(0.82–1.13)	0.640
≧30,000	0.82	(0.66–1.02)	0.075	0.86	(0.68–1.09)	0.219
Hypertension						
No	Ref.			Ref.		
Yes	1.97	(1.67–2.33)	<0.001	1.19	(0.99–1.43)	0.056
DM						
No	Ref.			Ref.		
Yes	2.53	(2.05–3.11)	<0.001	1.70	(1.36–2.12)	<0.001
Hyperlipidemia						
No	Ref.			Ref.		
Yes	1.82	(1.47–2.25)	<0.001	1.14	(0.91–1.44)	0.246
CVD						
No	Ref.			Ref.		
Yes	1.97	(1.43–2.72)	<0.001	1.13	(0.81–1.57)	0.463
Cerebral vascular disease						
No	Ref.			Ref.		
Yes	1.69	(1.31–2.19)	<0.001	0.95	(0.72–1.24)	0.688
Liver disease						
No	Ref.			Ref.		
Yes	1.29	(1.01–1.66)	0.045	1.04	(0.80–1.34)	0.793
Gout						
No	Ref.			Ref.		
Yes	2.64	(2.08–3.36)	<0.001	1.84	(1.44–2.36)	<0.001
Obesity						
No	Ref.			Ref.		
Yes	1.51	(0.38–6.05)	0.560	1.61	(0.40–6.45)	0.505
Depression						
No	Ref.			Ref.		
Yes	1.09	(0.78–1.49)	0.595	0.95	(0.69–1.30)	0.734

Adjusted age, gender, index year, urbanization, regions, monthly Income, visit ambulatory frequency, and comorbidities (hypertension, diabetes, hyperlipidemia, cardiovascular disease, cerebral vascular disease, liver disease, gout, obesity, depression).

### Cumulative incidences of CKD between NASD and control cohorts

**Fig 2:**
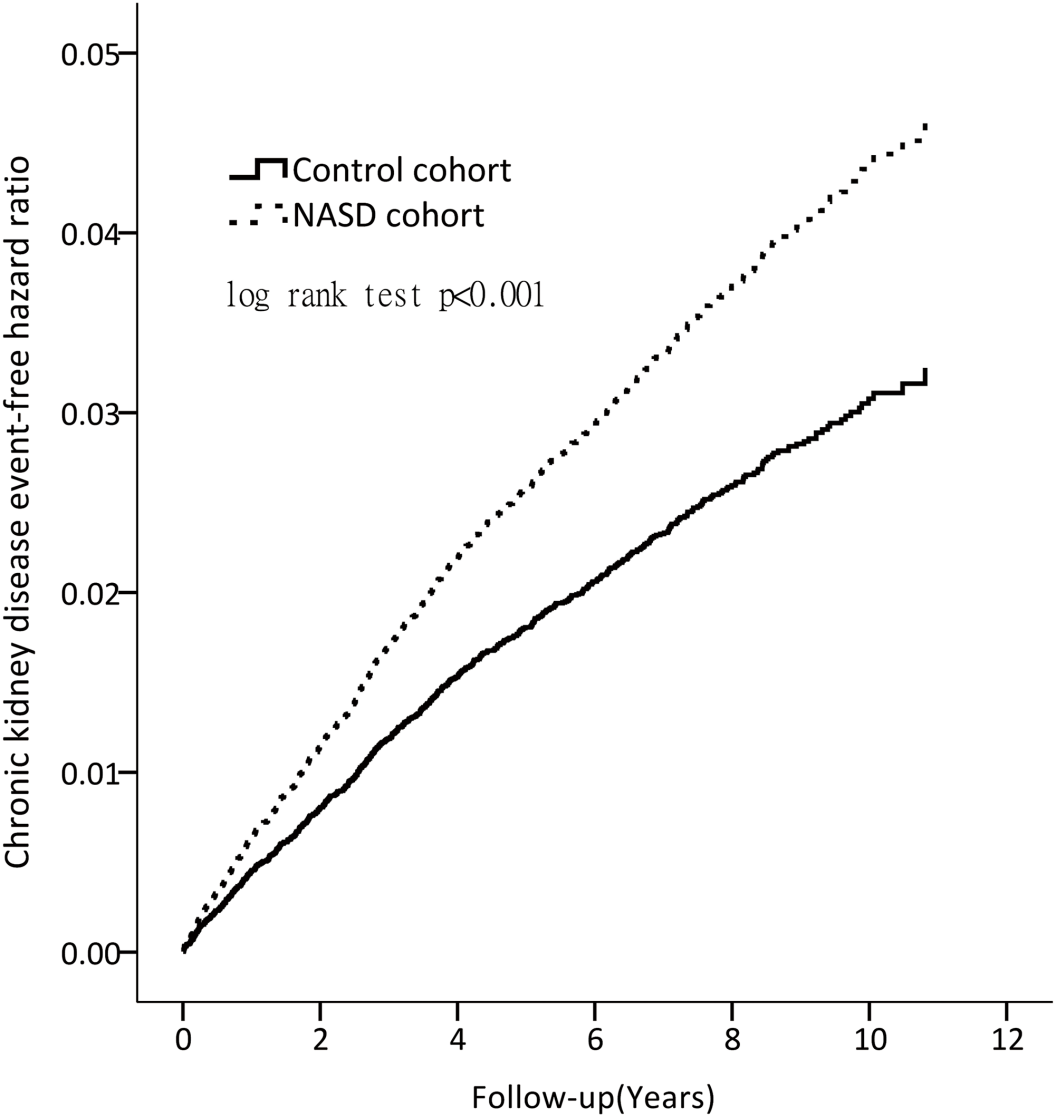
Cumulative incidence of CKD in the NASD (dash line) and control (solid) cohorts.

We further evaluated the cumulative incidence of CKD, and the risk of CKD was significantly higher in the NASD cohort than in the control cohort (10-year cumulative incidence, 3.77% vs.2.33%; 95% CI = 3.35%–4.24% vs.2.13%–2.54%; log-rank test, p< 0.001), as shown in Fig 2. For women aged <65 years, the 10-year cumulative incidence of CKD was 2.7% in the NASD cohort (HR = 1.81, 95% CI, 1.35–2.40) versus 1.5% in the control cohort. The 10-year cumulative incidence of CKD for men aged ≥ 65 years was 7.6% (HR = 2.27, 95% CI, 1.23–4.18, p = 0.009) and < 65 years was3.0% (HR = 1.49, 95% CI, 1.09–2.04, p = 0.013)in the NASD cohort versus 1.8% of< 65 years in the control cohort (Table 3).

**Table 3:** The risk of CKD development between the NASD cohort and control cohort stratified by sex and age(N = 28,024).

	<65 years old(N = 20,184)				≧65 years old(N = 7840)			
	No. Cases	(%)	Adjusted HR (95% CI)	p value	No. Cases	(%)	Adjusted HR (95% CI)	p value
Female(N = 15,744)								
Control cohort	129	(1.5)	Ref.		105	(3.4)	1.29 (0.60–2.80)	0.519
NASD cohort	79	(2.7)	1.81 (1.35–2.40)	<0.001	50	(4.7)	1.69 (0.76–3.77)	0.200
Male(N = 12,280)								
Control cohort	118	(1.8)	Ref.		138	(5.0)	1.74 (0.97–3.14)	0.064
NASD cohort	65	(3.0)	1.49 (1.09–2.04)	0.013	70	(7.6)	2.27 (1.23–4.18)	0.009

Adjusted age, gender, index year, urbanization, regions, monthly Income, visit ambulatory frequency, and comorbidities (hypertension, diabetes, hyperlipidemia, cardiovascular disease, cerebral vascular disease, liver disease, gout, depression).

### Subgroup analysis

**Fig 3:**
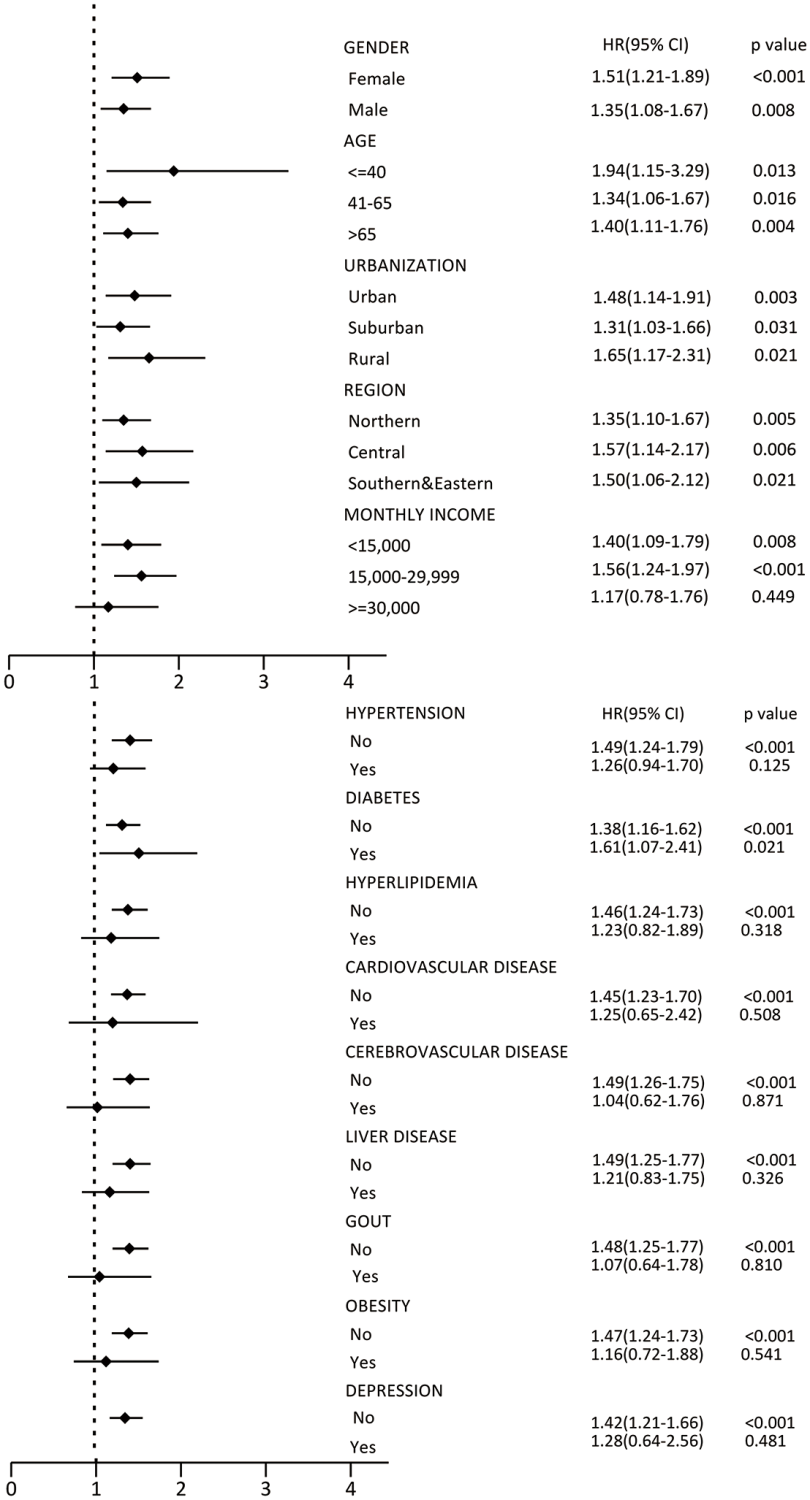
Forest tree plot of the HR increasing of CKD for (3a) baseline characteristics; (3b) comorbidities.

We further examined the association between the risk of CKD and subgroups of NASD. The risks of CKD were significantly in the NASD subgroups of insomnia (HR = 1.52, 95% CI, 1.23–1.84, p<0.001) and sleep disturbance (HR = 1.49, 95% CI, 1.19–1.87, p<0.001), but not in patients with other sleep disorders (HR = 1.00, 95% CI, 0.69–1.46, p = 0.985) (Table 4). The risk of CKD in different patient subgroups is shown in Fig 3a and 3b. After adjustment for variables, the risk of CKD was more prominent in patients in the following subgroups: female; male; aged≤ 40 years; aged 41–65 years, aged >65 years; living in urban, suburban, and rural areas; living in Northern, Central, Southern and Eastern Taiwan; monthly income < NT$15,000 and between NT$15,000 and NT$30,000; without hypertension; with and without DM; without hyperlipidemia; without CVD; without liver disease; without gout; without obesity; and without depression in the NASD cohort (p <0.05) (Fig 3a and 3b).

**Table 4:** Compared the risk of chronic kidney disease among different type NASD cohort. (N = 28,024).

			NASD cohort vs. Compared cohort		
	Case	per 1,000 person-years	Crude HR (95% CI)	Adjusted HR (95% CI)	p value
Control cohort	490	3.95	Ref.	Ref.	
NASD cohort					
Insomnia	138	7.13	1.80 (1.49–2.17)	1.52 (1.23–1.84)	<0.001
Sleep disturbance	97	6.53	1.65 (1.33–2.05)	1.49 (1.19–1.87)	<0.001
Other sleep disorder	29	4.17	1.04 (0.72–1.51)	1.00 (0.69–1.46)	0.985

Adjusted age, gender, index year, urbanization, regions, monthly Income, visit ambulatory frequency, and comorbidities (hypertension, diabetes, hyperlipidemia, cardiovascular disease, cerebral vascular disease, liver disease, gout, depression).

### Sensitivity Analysis

When alternative all-comorbidity-matching was applied, the association pattern was similar to that obtained in the aforementioned main analyses. As shown in S1 and S2 Tables, the risk of CKD was significant for patients with NASD (HR = 1.39; 95% CI = 1.15–1.69, p = 0.001).

## Discussion

We investigated the association between NASD and CKD. In this study, the risk of CKD was 1.48-fold higher in the NASD cohort than in the control cohort. An increased risk of CKD was observed in women younger than 65 years and in men of any age (men aged older than 65 years had the highest risk).

Few studies have investigated the association between NASD and CKD. Sleep disorder is highly prevalent in patients with CKD and ESRD [18,19]. Plantinga et al.[10] enrolled 9,110 participants (noninstitutionalized residents) in the United States and reported that sleep-related problems were more prevalent in patients with CKD. Agarwal et al.[20] compared the prevalence of sleep disturbances among patients with CKD receiving dialysis and those without CKD and reported that patients with CKD had lower sleep efficiency and higher sleep fragmentation, and sleep disruption in patients with CKD receiving dialysis was more severe than in those without CKD. However, the effect of CKD and its progression on renal function and various parameters of sleep quality revealed no significant linear pattern. Iseki et al.[21]recruited 5,651 people from the general population who received full-scale polysomnography, which was used as a diagnosis tool in Okinawa, Japan. OSA is more prevalent in patients with CKD than in patients without CKD. However, no cohort study has explored the association of NASD with the risk of CKD. In this cohort study, we used a large nationwide data set that afforded considerable statistical power and enabled long-term tracking of incident CKD events.

In our findings, patients in the NASD cohort had a higher risk of CKD than did those in the control cohort. Among patients with NASD, 47% of cases were diagnosed as unspecified insomnia, 35.2% were diagnosed as sleep disturbances, and 17.8% were diagnosed as other sleep disorders. In the NASD cohort, women (56.2%), patients older than 65 years (26.4%), and patients with lower monthly income (< NT$3,000, 83.1%) were predominant. The epidemiological results of this study were consistent with those of a previous population-based study in another country [22]. The prevalence of comorbidities was significantly higher in the NASD cohort than in the control cohort, and the risk of CKD was significantly high after adjustment for all baseline covariates. Older people were at a higher risk of sleep disorders and CKD than younger people in several studies [22–25]. However, in our subgroup analysis, younger women in the NASD cohort had a higher cumulative incidence of CKD compared with those in other subgroups in the control cohort (p<0.001). Additional studies evaluating the risk of kidney disease in younger female patients may be required.

Sleep and the circadian rhythm are fundamentally biological behaviors in animals and humans who are affected by pathology, stress, and personal habits, ultimately leading to unhealthy outcomes. Healthy sleep means optimal sleep quality and quantity. Sufficient sleep can improve general conditions, such as mood, alertness, and daily performance, and has beneficial long-term health outcomes. However, impaired sleep quality and quantity may be associated with increases in blood pressure (BP), the heart rate, inflammatory markers, and glucose intolerance [26–28]. In the ancillary of Coronary Artery Risk Development in Young Adults (CARDIA) study, which enrolled 578 adults aged 33–45 years in the United States, reduced sleep duration and consolidation predicted higher BP levels and adverse changes in BP [29]. Short sleep duration was a significant risk factor for hypertension in a study on the first National Health and Nutrition Examination Survey (NHANES I), which enrolled 4,810 patients with self-reported short sleep duration [30]. In the National Institutes of Health–AARP Diet and Health Study, which enrolled 174,542 participants in the United States, short night sleeps were associated with diabetes [31]. In a meta analysis on DM comprising 10 studies, the quantity and quality of sleep predict the risk of type 2 diabetes [32]. In a cross-sectional study that enrolled 1,688 people from 2 interlinked primary care databases in the United Kingdom, patients with gout were associated with any sleep problem (odds ratio [OR]: 1.39; 95% CI: 1.06–1.81) and, specifically, sleep problems other than sleep apnea (OR: 1.37; 95% CI: 1.03–1.82) [33]. The results of these studies may explain the possible pathological mechanisms of CKD among patients with NASD.

During sleep, reduced sympathetic tone and increased vagal tone cause the nocturnal decrease of BP. When people are in a sleep-debt condition, the sympathetic nervous system is activated [28]. Patients with CKD or ESRD exhibit dysregulation of the autonomic nervous system, manifesting as a failure to increase the heart rate variability [34] and leading to a dip in BP [35]. The activated sympathetic nervous system may contribute to the pathogenesis of renal hypertension and is postulated as a risk factor for CKD [36]. Circadian rhythms are linked to human homeostasis and BP. The oscillation of the renin–angiotensin–aldosterone system is modulated by the circadian rhythm and rapid eye movement (REM)–non-REM cycle [37,38]. Total sleep deprivation in patients with depression leads to an increase in renin secretion and a concomitant trend for a decrease in the hypothalamic–pituitary–adrenal axis activity next night [39]. An alteration in the activity of the renin–angiotensin–aldosterone system could be another pathological mechanism of the risk of kidney disease. Sleep curtailment increased the level of proinflammatory cytokines, high sensitivity c-reactive protein and white blood cells [27,40]. In Sauvet et al. [41], vascular dysfunction was observed before an increase in sympathetic activity and systolic blood pressure with sleep deprivation. In Ohkuma et al.[42], the urinary albumin-creatinine ratio, which is a biomarker of kidney function, was associated with sleep duration in patients with type 2 diabetes. Inflammation causes glomerular endothelial dysfunction, which may lead to renal function decrease.

Huang et al. evaluated the incidence of NASD and CKD from the NHIRD and reported a significantly increased risk of CKD in patients with NASD[43]. The differences between their study and ours are the definitions of NASD and CKD, presence of comorbidities, subgroup analysis, and prognosis evaluation. The major strength of our study is that it was designed to reduce selection bias with the random sampling of a large nationwide population-based and highly representative sample, to limit detection bias of considering the use of medical services, and to mitigate environmental effects according to availability of socioeconomic indicators for all participants. Our findings of an increased risk of CKD in patients with NASD are robust because the study population was well defined and the follow-up was complete.

### Limitations

Our study had several limitations. First, the lack of data on objective sleep measurement or other mental health conditions that are highly comorbid with NASD is a critical limitation. Second, diseases may be inaccurately classified when an administrative database is used. To counter this concern, NASD was diagnosed according to both ICD-9-CM codes and benzodiazepine use. Moreover, the NHI reviews patient charts, audits medical charges, and imposes heavy penalties for inappropriate charges or malpractice to ensure claim accuracy. Third, the comorbidities of NASD were significantly higher in the NASD cohort than in the control cohort. Therefore, we further performed a sensitivity analysis to match comorbidities from both cohorts, and the results were positive. Fourth, because of the definition of NASD in our cohort, the effect of the risk of CKD from BZD use requires investigation. We analyzed the BZD dose response with the risk of CKD and the results were negative. Fifth, the NHIRD lacks information on body weight, laboratory data, lifestyle, and family history of kidney disease; all of which may contribute to the risk of CKD. Therefore, these variables could not be included in the propensity analysis because adjustment could not be performed, leading to the difference in propensity scores between the cohorts. Therefore, we added the CCI score to the propensity score in multivariate and stratified analyses to control for confounding by these variables. Finally, NHIRD is a disconnected research database. The unknown of symptom period of NASD may cause an underestimation of the incidence of CKD in patients with NASD. Despite these limitations, this study had several strengths. This was a longitudinal nationwide population-based cohort study on an Asian population regarding the association between NASD and the risk of subsequent CKD events. Our findings may benefit further analysis in future studies regarding specific sleep disorders contributing to CKD incidence.

### Conclusion

In this Taiwanese nationwide population-based cohort study, NASD was significantly associated with increased risks of CKD, particularly in men and women younger than 65 years. Enhancing sleep disorder management may bevital for CKD prevention because of the increase in the number of patients with NASD.

## Supporting Information

S1 Table Demographic characteristics between the NASD cohort and control cohort matched with sex, age, index year and comorbidities with 1:1 ratio. (DOCX)

S2 Table Comparison of the risk of CKD between the NASD cohort and control cohort. (DOCX)
