# Supplementary material for: Nonapnea Sleep Disorders in Patients Younger than 65 Years Are Significantly Associated with CKD: A Nationwide Population-Based Study
Source: PLoS One. 2015 Oct 14;10(10):e0140401. doi: 10.1371/journal.pone.0140401 (PMC4605694; doi:10.1371/journal.pone.0140401)
Supplement: S1 Table — (DOCX) [file pone.0140401.s001.docx]

| S Table 1 Demographic characteristics between the NASD cohort and control cohort matched with sex, age, index year and comorbidities with 1:1 ratio (N=14,010) | | | | | |
| --- | --- | --- | --- | --- | --- |
|  | NASD cohort  (n=7,005) | | Compared cohort  (n=7,005) | | *p* value |
| Age(Mean±SD) | 53.91 | (±16.45) | 54.05 | (±16.78) | 0.614 |
| ≦40 | 1688 | (24.1) | 1641 | (23.4) | 0.478 |
| 41-65 | 3466 | (49.5) | 3458 | (49.4) |  |
| >65 | 1851 | (26.4) | 1906 | (27.2) |  |
| Gender (%) |  |  |  |  |  |
| Female | 3935 | (56.2) | 3956 | (56.5) | 0.721 |
| Male | 3070 | (43.8) | 3049 | (43.5) |  |
| Urbanization |  |  |  |  |  |
| Urban | 2520 | (36.0) | 2516 | (35.9) | 0.997 |
| Suburban | 2917 | (41.6) | 2919 | (41.7) |  |
| Rural | 1568 | (22.4) | 1570 | (22.4) |  |
| Region |  |  |  |  |  |
| Northern | 3793 | (54.1) | 3786 | (54.1) | 0.991 |
| Central | 1680 | (24.0) | 1681 | (24.0) |  |
| Southern | 1286 | (18.4) | 1285 | (18.3) |  |
| Eastern | 246 | (3.5) | 253 | (3.6) |  |
| Monthly Income |  |  |  |  |  |
| <15,000 | 2563 | (36.6) | 2572 | (36.7) | 0.982 |
| 15,000-29,999 | 3260 | (46.5) | 3249 | (46.4) |  |
| ≧30,000 | 1182 | (16.9) | 1184 | (16.9) |  |
| Comorbidities(%) |  |  |  |  |  |
| Hypertension | 1410 | (20.1) | 1406 | (20.1) | 0.933 |
| DM | 569 | (8.1) | 558 | (8.0) | 0.733 |
| Hyperlipidemia | 784 | (11.2) | 784 | (11.2) | 1.000 |
| CVD | 277 | (4.0) | 257 | (3.7) | 0.378 |
| Cerebral vascular disease | 595 | (8.5) | 566 | (8.1) | 0.374 |
| Liver disease | 693 | (9.9) | 686 | (9.8) | 0.843 |
| Gout | 387 | (5.5) | 362 | (5.2) | 0.348 |
| Obesity | 30 | (0.4) | 29 | (0.4) | 0.896 |
| Depression | 908 | (13.0) | 903 | (12.9) | 0.900 |
| Abbreviation: NASD: nonapnea sleep disorders; SD: standard deviation; DM: type 2 Diabetes Mellitus; CVD: cardiovascular disease; COPD: chronic pulmonary disease  The difference of two cohort was estimated by independent t test or chi-square test. | | | | | |
