# Supplementary material for: Nonapnea Sleep Disorders in Patients Younger than 65 Years Are Significantly Associated with CKD: A Nationwide Population-Based Study
Source: PLoS One. 2015 Oct 14;10(10):e0140401. doi: 10.1371/journal.pone.0140401 (PMC4605694; doi:10.1371/journal.pone.0140401)
Supplement: S2 Table — (DOCX) [file pone.0140401.s002.docx]

| S Table 2 Comparison of the risk of CKD between the NASD cohort and control cohort.(N=14,008) | | | | | | | | |
| --- | --- | --- | --- | --- | --- | --- | --- | --- |
|  |  | | |  | NASD cohort vs. Control cohort | | | |
|  | Case | person-years | Rate^**^ |  | Crude HR  (95% CI) |  | Adjusted HR  (95% CI) | p value |
| Control cohort | 177 | 38399.32 | 4.61 |  | Ref. |  | Ref. |  |
| NASD cohort | 264 | 41136.18 | 6.42 |  | 1.40  (1.16-1.70) |  | 1.39  (1.15-1.69) | 0.001 |
| * Adjusted age, gender, index year, urbanization, regions, monthly Income, visit ambulatory frequency, and comorbidities (hypertension, diabetes, hyperlipidemia, cardiovascular disease, cerebral vascular disease, , liver disease, gout, obesity, depression)  ^**^ Rate: Per-1000 person year. | | | | | | | | |
